# Children’s Rights

**Published:** 1893-07

**Authors:** I. N. Love

**Affiliations:** St. Louis


Buffalo Medical and Surgical Journal

VOL. XXXII. JULY, 1893. No. 12.

### Original Address.

### CHILDREN'S RIGHTS.

VALEDICTORY ADDRESS DELIVERED BEFORE THE MARION-SIMS COLLEGE OF MEDICINE, MARCH 23, 1893.

BY I. N. LOVE, M. D., St. Louis.

IT IS my desire to bore you but a little while. Time out of mind, valedictory addresses have been delivered. The question which has presented itself to my mind occasionally since this duty was assigned me, has been, "Of what shall I speak?" Surely, the entire field has been covered over and over again. The subject least often considered on such an occasion, for the reason, possibly, that it seems insignificant rather than formidable or dignified, is the one which I have chosen,—namely, "Children's Rights;" and in the beginning, I can only say that I regret in the extreme that the strong-minded women of the world have not concentrated their great intellects upon the consideration of the amelioration of the wrongs of children rather than those of the female sex, because, really, women's wrongs only exist in the minds of these agitators. Bless their souls! have not women all the rights that any one could ask for? The right of being loved early and often; the right of being slaved for; the right of being protected; the right of being sought for; the right of being the dearest, sweetest, noblest, and best creatures, more closely in touch with man and Heaven than anything on the earth today? What more do they want? They surely have the earth already.

But the children—what are their rights? I grant that more time, thought, money, and effort are being directed toward the improvement of children today than ever before in the history of the world, and yet it is not enough.

Time was when the only right seemingly granted to children was the right of being born, and to this day, this right is granted them without consulting their comfort or wishes. They are fully justified often in asking the question, "What are we here for?" Surely, in response to the selfish desires of the physical authors of their being. The changes have been rung for centuries in monotonous regularity, upon the great obligation which children are under to their parents for bringing them into a world of suffering, care, worry, and death. There are many who still think that the only right of a child is to be born, or by those puritanically inclined, it may be granted the additional right of being born again or being damned forever. All will grant the truth of Tupper's announcement that "a babe in the house is a well-spring of pleasure"; at least, if it is not, it should be. Is there anything more beautiful, more sweet, more lovable, more heavenly, than a baby? Nothing, unless it be two babies! Yes, truly, a baby is a thing of beauty, and we have good authority for the statement that it is a joy forever; but are babies developed in the direction of being perpetual joys? Does their loveliness increase? Will they never pass into nothingness, or, what is worse than nothingness, cussedness? As the statement has often been made, that the problem as to whether life is worth living depends much upon the liver, so we are safe in saying that the question as to whether a babe is worth the borning and all that the term implies, depends much upon those engaged in and responsible for the borning. I make bold to say that the men who have invested their money and given their gray matter towards the raising of the finer breeds of horses, cattle, and other of the lower animals, manifest greater intelligence and appreciation of their awful responsibilities than do the average parents. Unquestionably, one of the greatest obstructions today to the proper appreciation of duty on the part of parents to their children, is due to the maniacal struggle for money and place. Fathers work frantically day and night for gold with which to buy position and power. Mothers, too many of them, are filled with the desire for social preferment or the securement of that place in society which will enable them to outshine some one else. Both father and mother should be satisfied with a less degree of success in these directions and determine in the beginning of their career to work towards the furnishing to the State of high-bred, good-blooded children. There is one thing in the Catholic church which commands my admiration, and that is their teaching in the direction of duty to the home and children, particularly upon the part of mothers; in fact, I am strong in the belief that the world owes much to the Catholic church for its noble fight for the rights of motherhood, for the nobility and dignity of motherhood. It might well be called the "Mother Church," if for no other reason than that of having placed the Virgin Mary upon the level of God himself, thus placing maternity close to divinity.

Don't understand me to announce the belief in the thought that there are many women in the world devoid of the maternal instinct and who wilfully neglect their children. The reverse proposition is true. Even the petted darlings of society who develop into mothers, are not devoid of the strongest mother-love. The charge that the society woman is disposed to knowingly neglect her child, particularly when its life is involved or the question of furnishing nourishment to it is presented, is, in my judgment, not true. I have never met one woman who wilfully declined to perform her duty when the question of the life or death of her child was presented to her.

The statement has been made that the masculine mind makes its blunders by overlooking details and the feminine by seeing nothing else, and yet I am confident that the blunders of motherhood are made owing to the fact that the mother fails to realize the importance of details of the little things connected with the well-being of her child.

That fathers are far more neglectful than mothers of their duty to children, all will admit. Too many fathers have indulged in the "eating of sour grapes and have thus set their children's teeth on edge." I grant that it makes very little difference, except from a physical point of view, who a child's father may be, but it is surely of vital importance as to the mother. We have only to look for the origin of the great men of the world and we shall see that they owe their greatness and their goodness, in the majority of cases, to good, true, noble mothers ; and yet might they not have been as good or even better had they had fathers as well equipped for paternal duties as the mothers ? No doubt, these sons developed well as the result of maternal work and training, but might not the burden have been lighter for the mother, and might not the result have been more satisfactory all around, had the father done his work as he should have done ? Surely, the fathers would have been happier in the possession of their children, had they been fully impressed with their duty to them. Too many fathers are disposed to think that if they struggle and fight and keep the wolf from the door, and even secure for their families a goodly quantity of "filthy lucre" (all of which possibly helps to make their children less strong, less self-reliant and able to cope with the dangers about them), that they have done their duty.

It is the right of every child to have something more than the privilege of calling a man father. It is his right to have that father realize that " filial affection, like patriotism, must be engrained as an obligation ; a thing to blush at if not possessed." If, however, the father absorbs himself only with money-getting and the fumes and frets of trades, professions, or the commercial world during the earlier years of the child's life, need he be surprised later in life to find that he knows not his own nor does his own know him?

The father calls the child into being. Having done so, he assumes a duty which he should not shirk. It is the right of every child to have something more in his father than a mere provider of food and raiment. Does the father want the honor, the respect, and the enjoyment of the sweetest part of life, he must needs make himself acquainted with his child from the day of its birth on. It is as much his duty to study the disposition, character, and general human nature of his child, as it is the mother's ; indeed, he has no more right to shirk his part of the sentimental relations between parent and child, than has the mother. Of course, the father who neglects the material interests of his children, on the theory that what was good enough for him is good enough for them (as has been well said by Pike in the *Century*), "should pay conscience money to his own father." All children have the right to claim that their parents give them the benefit of the mistakes made in raising them ; in truth, parents might well take pointers from horse, bird, and dog-fanciers, and cultivate themselves in the direction of becoming child-fanciers.

I am sometimes inclined to believe that if the children of the world could be consulted, those that have been and are to be, would be favorable to the doctrine set forth by Malthus in his essay on the Principle of Population, favoring the limiting of their number by the discouragement of early marriages and by the practice of moral self-restraint. The state at large would certainly be improved by having the population made better rather than more numerous. The family would be in better form if this rule obtained.

I think the lion had the better of the argument, when chaffed by her neighbor, the hare (possessed of a numerous offspring), on account of the meagerness of her progeny in point of numbers, when she responded, "But mine are lions!" We will desist, for we might soon get into deep waters when discussing the rights of children from this standpoint. Whether the family be composed of many or few, the fact still remains that the child should have the right to claim that his physical, moral, and mental necessities be looked after.

I think it was Oliver Wendell Holmes who once said that the time to treat some of the ills—mental, moral, and physical—of children would have been to have treated their great-grand-parents.

Children have a right to ask before being born that their parents be healthy. A common sense view of this question should be taken. The State should enact laws preventing the marriage of the criminal, or those diseased mentally and physically, and all who have reached the age of sixty. The child has a right to demand, if such marriages do take place, that it be protected; that preventive measures be taken to guard it against the transmission of unfavorable germs. With the existing knowledge that we have, the child should demand protection against disease in advance of its birth and even of its conception.

Knowing that each and every one of us is a part of all that we have met, we should realize that the child has a right to be protected from improper associations, whether these associations be the germs of diphtheria, scarlet fever, typhoid fever, or worse, the germs that are associated with vice and crime. An authority high in the church has given expression to the thought that if children can be kept under the church influence until the age of seven, they are secured in their faith through all coming time. How many parents realize this fact? The child has a right to demand protection from association with vicious children and ignorant adults. It seems to me that the parent should be jealous of every hour in the early life of his child. Without interfering with romping, playing, and the proper muscular exercise, the parent should be with the child a sufficient length of time each day that passes so as to greatly influence the bent of his mind. The cases are numerous and met with every day, where children with a depraved physical inheritance have been built up out and away from their inheritance into strong, robust, and healthful people. We all know now that which we did not know formerly, that food, in an available form, made into good blood, is the enemy to all the microbes of disease. The medical profession at large has long since been impressed with the importance of the teachings of Graves, the great Irish physician, and in the value of his words, when he said: "Let there be placed upon my tomb as an epitaph only this: 'He Fed Fevers.'" Koch's bacillus of consumption will, in every case, go to the wall, retire defeated from the field, if met with good rich blood. Rickets, scrofulosis, and all such can no longer hope to cope with the elements of nutrition. Weak, nervous systems, weak minds in children, even to the point of idiocy, are now being fed and furnished with strength. If this be true, and who will deny it, what cannot we hope to accomplish in the way of developing our children, mentally and morally, if we take our cue from those who demonstrate the possibilities of physical development.

Children have a right to demand that the same intelligence be directed toward their mental and moral development as toward their physical good. When we realize that seventy-five per cent. of the deaths that occur among children under five years are preventable, and directly due to errors of diet and improper clothing, we can understand how important that the child from the day of its birth be fed with proper food, in proper quantities, at the proper time, and be properly protected against exposure and chilling of the surfaces by proper clothing. Indeed, if the child can be physically fed, and it can be, long before it is born, is it not our duty to do it? The child that does not die from a cause directly produced by cold or improper food, is placed in a position to be more susceptible to the disease germs ever scattered broadcast in our midst. The illy-fed, the poorly-clothed have a starved nerve system and an impaired equipment, so that their power of resistance is lessened.

Do we expect to protect our children from disease by keeping them in band-boxes or glass cases? No! If we succeed in developing the child up to the age of seven, strong and well, the chances are that he will live a long life and a healthy one. The same reasoning will apply to the child from a mental and moral standpoint. The Klebs-Loeffler's bacillus of diphtheria, the typhoid germ, and the comma-bacillus of cholera, are all bad enough and gravely dangerous to the child, but how much more deadly the two-legged bacillus, the product of the culture of the slums of "Kerry Patch" and "Clabber Alley," to whose constant care the children of some parents are entrusted, in apparently blissful ignorance of the fact that they are moral lepers whose souls are festering in filth to the point of putridity and even gangrene.

An atmosphere of cheerfulness should ever be present in the home. The cares of business should not be echoed and reflected within the reach of the ears of children. Truly, the sad and sorrowful should be kept from them as a rule, and yet a proper knowledge of all these things should from time to time be allowed. It is true in childhood as it is later in life, that everything in the world may be surely and safely enjoyed except continual prosperity. The refining and improving effects of disappointment and an occasional sad hour should be considered.

The child has a right to be prevented from becoming disobedient. Many are taught to be so by thoughtless mothers. When Johnny is informed that he is not to do a certain thing or things, and that if he does he will be severely punished; and Johnny thoughtlessly disobeys and the disobedience is unnoticed, what is more natural than for Johnny to conclude that mother does not mean what she says, and how natural for Johnny to put little stress upon her injunctions. How surprised he is to find later that his errors receive attention and possibly a hasty and unkind punishment.

In this connection, permit me to say that I think that no child, if properly managed from the beginning, need ever be whipped; in fact, there should be a law prohibiting the whipping of a child by any one, teacher or parent, with a penalty attached for its infraction; said penalty to be a thrashing at the hands of a public whipper. I would favor confining the whipping post to this class of offenders.

An old Chinese proverb says that "rogues differ little; each begins first as a disobedient son," and it might well have said that each disobedient son was educated in the direction of disobedience by inconsistent parents. Business men whose checks can be received for any amount of money, and whose promise to pay would be considered as good as gold, often forget their word to their children, from the beginning of their existence. The commercial standing of the business man is probably at stake and has a money value to him, and this has much to do in developing his high sense of business honor; but it should be of greater merit to a man to rank high as a parent rather than as a business man.

The child has a right to be born into a home where parents realize that the home, after all, is the chief part of life; where the father is not so busy that he has no leisure to laugh, and the whole business of his life seems not to be to get money, more money, and yet more money, that he may still get more. Such a father should recall to his mind the text: "For what does it profit a man if he gains the whole world and loses the satisfaction of having about him healthy, happy, merry children." Children are born honest and cheerful, with a capacity for happiness—all just as natural to them as good digestive organs, and an appetite for food. The child that becomes dishonest, misanthropic, suspicious, gloomy natured, has often been made so by the treatment which he has received. The child should be granted, if I had the arranging of things, the privilege of selecting the parent and the teacher best adapted to it; and, by the way, the teacher is almost as important to the child as the parent. Our present school system is defective in many ways; chiefly in that the highest priced and best teachers are not the ones in charge of the smaller children. Teachers, like nurses, are born, not made. The individual who does not love children; who is not in sympathy with them; who is not patient, gentle, kind, honest, sincere, trusting, cheerful, and the complete ruler of his own spirit, should not be placed in charge of children.

The kindergarten is all right, except that it serves in some cases as a hot-house to over-develop an already rapidly growing brain. The bright child needs but little kindergarten training. Another objection is that at the tender age, the child is taken from the society of its mother, weaned as it were, and placed to the breast of a stranger, a very proper person, no doubt, and yet not its mother. Long years ago, Hosea Bigelow said in his quaint way: "For it strikes me ther's such a thing ez sinnin' by over-loadin' children's under-pinnin'." There is, indeed, too much over-loading. Many children, before they arrive at the age of ten, the time at which they ought to start to school, are in the position of the spavened and wind-broken thorough-bred which is put upon the turf too young. The old-time school, built in many instances of logs, with numerous openings, permitting free ventilation, situated often many miles away from the homes of the children, necessitating long walks and but few hours in school, seemed inefficient viewed from this distance; particularly did the sessions seem short, being in the majority of instances only three months in the year, but a good lusty mental and physical development was the result. The school year is too long now. It should be cut off at both ends, making the session begin in October and close in May. The discipline in the old-time school was severe, but its course of study less brutal than today. The rod and the various forms of punishment were all wrong, but children did not often die of the severe treatment received from the old masters, though no one knows better than the doctor that many succumb to the hard requirements of the modern methods. Nervous, sensitive children suffered intensely from the old-style discipline the same as they did from the old severe regime practised in the home. The puritanical way of looking at things was not always the pleasantest. He was a very correct man, viewed from the standpoint of the early rock-ribbed creeds, who admonished his children: "Children, love one another, confound you." But where there was a single delicate one demoralized and injured, sometimes irreparably by harsh rules and customs of those early days, there are a hundred that suffer now from mental stuffing and brain fag. No one would think of placing fifty or a hundred children, either of small or larger growth, at a table with a uniform bill of fare, commanding all to eat the same amount of the same food, good, bad and indifferent, without looking for numerous cases of indigestion and even fatalities to follow; but in the schools this thing is done every day. The child's capacity for digesting this subject or that branch of knowledge, is not considered. Of course, more thought is given to the subject than formerly, but not enough. It is true that the supply of money is often inefficient to make the schools ideal, but the expense could be diminished in many directions and improvements made to follow. First of all, our public school system has no right to pursue a course which educates the few rather than the many. Manual training, both for boys and girls, should be engrafted upon our public school system. The number of studies should be lessened; the time shortened; the teachers should be made to teach. This duty falls to the mothers now largely. The mothers teach their children the lessons, and often wear themselves out in the effort, and the child goes to school merely to recite. More attention should be given to reading, and a part of the time of other branches should be given up to reading interesting matter. If all of the school studies were secondary to habits of good reading, the child would be taught at school as he should be, to be a careful, discriminating reader through life. The child should be taught how to think and to express itself. English composition should be encouraged. I am surprised to see how little attention is given of late to penmanship. The teacher who studies the individuality of the child and gets closely in touch with it, and encourages conversations pertaining to the subjects under consideration, will be most successful; and, by the way, the rule adopted some time ago by our school board, excluding married teachers, is ridiculous and absurd. Other things being equal, the married teacher, particularly if she has had children, is the better adapted to understand children and to know their needs. She is in every way better equipped for the work before her than the young unmarried woman, however well educated she may be. The latter, nine times out of ten, takes the position merely to give her an income which will enable her to get married. The married teacher is in the work because she loves it, or from necessity ; and the chances are that she desires to remain for life. What business is it to the school-board if she is so unfortunate in some cases as to get a worthless husband who cannot make a living for her ? This is her misfortune and the schools are in no way affected, except to be benefited ; and, besides, at the present rate of progress in the dislocation of the sexes, the crowding of women into the avocations naturally belonging to men, what are men to do unless they can marry some of the women who have crowded them out, and get food and raiment in this way ?

Children have a right to be treated at school and at home, generously, kindly, tenderly, patiently, honestly, and above all, justly. Children are generous in their enthusiasms ; they are not deaf to wit, but, perhaps, of all other things, they admire justice most. The sense of humor in children should be encouraged and developed, because the one who grows into manhood without it, loses much of the zest of life. The boy or the man who cannot take a joke is unfortunate. The boy who responded to his teacher when asked how it was that there was no W in the French language, by saying, because of Waterloo, and was given a black mark for his answer, was more fortunate than his teacher, for he was possessed of a keen sense of wit and his teacher was devoid of an appreciation of it. Yes, cultivate the spirit of fun. Truly, if a "little fun now and then, is relished by the best of men," it should be relished by the child, and if it is not relished, there is something the matter with the child. There was a good deal expressed in this verse from The Boys, by Oliver Wendell Holmes :

> You hear that boy laughing, you think he is all fun ;
> But the angels laugh too at the good he has done.
> And the children laugh loud as they troop to his call,
> But the poor man who knows him, laughs loudest of all.

Children have a right to be taught that rare talent that grows rarer as the years pass,—the talent of appreciation. They should be taught to know their blessings, their good fortunes, and the obligations under which they rest, not only to their parents but to their fellows. They should be taught how to render service and accept it; render kindnesses and accept them; to grant obligations and to accept them; and to do both gracefully.

Children have a right to demand the completed cultivation of one of the most important organs given to them,—namely, their voice. They cannot all be made good singers, but the voice of every individual child can be developed in the direction of being beautiful. I believe that nowhere on earth is the human voice more neglected than in America. The number of shrill, harsh-voiced girls that one comes in contact with daily is appalling. Too often not sufficient attention is given to the voice, and the habit of loudness and carelessness of speech is developed. The girl has been badly educated, and the boy as well, no matter what their accomplishments, unless they have learned that the secret of being understood lies more in enunciation than loudness. There are many nerves being wounded and irreparably injured each day that passes, by the beastly harshness and careless roughness of uncultivated voices than are being worn out by over-work or whipped out by alcohol. It will be difficult for one who ever saw the beautiful, angelic Adelaide Neilson, to forget her beauty, but no one who was fortunate enough to have heard the gentle, soothing notes of her lute-like voice will ever forget them. I think that the faithful one in dying, as he approaches the gates of Heaven, may expect to hear notes reminding him of the tones of that voice. Is there anything that is more lasting and that more frequently comes back from the way beyond, to the ear of a boy, even though he has reached his second childhood, than the gentle echoes of a sweet-voiced mother? I recall a big, strong, rough-and-ready man to my mind, who, when dangerously ill, referred to his mother long since dead, and the strongest point he made in her favor was that she was a gentlewoman and so kindly voiced as always to have had an influence over him in life beyond that of anything on earth; and that the memory of her sweet voice came to him ever and often when tired, weary, and worn, and gave him inspiration.

Gentlemen of the graduating class of 1893, the time has arrived for the Faculty to say good-bye to you, and it has fallen to my lot to say it. I know that I voice the sentiments of my associates when I say that every hour that we have passed with you has been a pleasant one. You have been earnest, honest, faithful students. We have endeavored to be the same, as teachers. We ask that you continue to be as you have been during the time that you have been with us. Continue to be students during all the years of your lives. Remember that the best medical college on earth is nothing more than a kindergarten. Remember that the ablest man that ever lived, that the strongest brained and the sturdiest bodied, could not do his full duty to the profession of medicine; could not return to it a tithe of that which he owes it and receives from it, were he to work every hour of the twenty-four during all the years of his life. The ablest man cannot hope to do his full duty, but the weakest of us can strive in the direction of duty. This class may not furnish even one of the few immortal names that were not born to die, but let us hope that it will furnish a set of men who will never forget the obligations they have assumed. The chief dangers which surround the doctor in this life, are the demons of laziness and fatigue. When unemployed, laziness may take possession, and as time passes and success has crowned your efforts in the direction of becoming established, and you become a thoroughly busy man, the danger of fatigue, physical and mental, will be ever present, and this danger you must fight against. If yielded to, you will cease your studies and seek rest instead, and soon you will have formed the habit of neglecting your books and the recorded observations of workers in the field of medicine.

Never lose sight of the importance of guarding the health of your bodies. A half sick doctor is no doctor at all. The only thing for a doctor to do if broken down, is to quit the profession or die ; therefore, guard against these break-downs.

A most important thing for you is the selection of your location. Take plenty of time to do this. Look the field over well, and when once located, let it be for life. A rolling stone gathers no moss, and there are more rolling stones in the medical profession than in any other on earth. Too many doctors change their locations too often. The mistake was made originally in not taking plenty of time in the selection. On general principles a good man does well to locate where he is already known, even where he has grown up. While it may be true that a prophet is not without honor, save in his own country and in his own house, that thought does not apply to doctors, for they are not prophets. It matters little whether you select the city or the country, as the work before you is a noble one. The doctor may well say with the prophet of old, "Give me neither poverty nor riches;" because if he does his duty, that is about what he will get anyway. The Good Book must mean doctors when it says that "he that maketh haste to be rich, shall not be innocent." Be of good courage, for just as sure as the day comes after the night, if you follow in the lines which you have laid out, success will come to you. If thee faint in the day of adversity, thy strength is small. After all, it is not adversity that tries men most. It is prosperity. Your severest struggles will come after your success has come; and, by the way, you will often find that prosperity is the severest test not only of yourself, but of your friends. Be not afraid to cast thy bread upon the water, for even though it be many days before it returns, it will surely return, and, more than likely, draw with it loads of fishes.

Be gentle, kind, good, patient, and faithful to your patients, but do not expect them to be so to you. You will find that not only Republics are ungrateful, but patients as well. Your tastes, associations, and bringing up may carry you in relation with church and society work, and possibly in the direction of one or the other of the political parties; but my advice to you is to keep in mind the words of Solomon, "Be not righteous, over much." I know men who are better Presbyterians than they are physicians. The medical profession is a jealous mistress. Concentration is necessary to a proper understanding of your work, and while never losing for one moment sight of the necessity, if you would be happy now or hereafter, of having faith in the living God, let other people in the community run the churches. Let your chief prayer-book be a medical text-book; let your prayer meeting be the medical society.

You will find in every community one or more members of the profession who seem to be washing their hands with invisible soap in imperceptible water; who are sanctimoniousness personified; who work the church for all there is in it; but let them do it. You can well afford to follow the thought that people want a doctor for his skill and not because he tries to walk their particular chalk-line.

Among the many annoyances with which you will have to contend, quacks and mountebanks will abound. The honest quack who takes the page in the local paper and prates about his skill, is not the most annoying. Of the general quack, I think the most contemptible is the one who would sneak into a practice under the mantle of the meek and modest one; under the banner of the bleeding blessed Jesus. But to all of them, give naught but indifference. Above all things, do not dignify them by discussion. Be patient and tolerant with such, and remember that they are so partly because they were built so and partly because association and training were at fault. Do not certainly dignify the irregulars of the profession by discussing them with the laity. Remember that there can be no sectarianism in medicine, and, while admitting that there are more ways than one of skinning a cat, grant to any and all the privilege of skinning their particular cat in their particular way. Argumentation on such matters, is time lost. I agree with Tristem Varick that there is no use in arguing with any one. It is talking metaphysics to a bull. The first thing you know, you get a horn in your navel. Do not cultivate local fraternal societies. Membership in them is all right, but let it not be at the expense of your time. There are doctors who know more of masonic matters than medicine, and the public may safely avoid them. Each doctor should take his place in the community and bear his part in the duties of citizenship and social life. Every doctor should vote and if needs be use his influence for the best man, but I implore you, avoid politics. A political doctor, manipulating caucuses, presiding over party central committees, directing and controlling the "hoodlum" vote of cities, large or small, runs the risk soon of being known more as a bummer or a boodler than as a scientist or a friend of humanity, and is a sight sufficient to make the spirits of Hunter, Harvey, Ephraim McDowell, Hodgen, and Gross become jangled and out of tune. Join yourself to no party that does not carry the flag and keep step to the music of the sciences.

So far as social life is concerned or matters pertaining to society, do your duty and your social position is fixed. You need envy no Mr. Moneybags on earth, because, after all, a man's place in this world is most secure who makes the most pronounced record of doing good. The true aristocracy is the aristocracy of usefulness and brains. You cannot hope to be a social success and a good doctor. The dear old autocrat of the breakfast table has said that "no fellow can be a thorough-going swell unless he has three generations in oil; mind you, daguerrotypes won't do." The only time that a doctor can cut a swell is when he lances a boil. You cannot hope to be a social success even were you to attempt it, except at the expense, at the absolute sacrifice of your profession and the best interests of your patients. I advise you to avoid society, for within its domains nimble heels are of more value than brains. If you doubt it, ask Ward McAllister. "It is a despot, and those brought into its courts are slaves." Fortunately for you, a doctor is not only an exception when it comes to a question of jury service, but he is an exception to all the rules of good society; and, by the way, what a whited sepulchre this same society is. It may, indeed, be beautiful outwardly, but who knows better than a doctor that within it is full of dead men's bones, and fuller of the bones of lovely women who have given themselves as sacrifices to its demands.

Remember always that a man's best interests are close to him and lie close about his feet; that "he most lives who thinks the most, feels the noblest, acts the best." Work to develop yourselves in an all-around way. Have your little recreations and hobbies if need be, but let them be merely incidental and only with a view to your best good. Keep in touch with general literature. Your mind will be brightened and you can better grasp the technicalities of your profession. Develop the poetical side of your nature. It will be of much pleasure to you in your dreary round of work. It is Lytton, I think, who says: "Poets are all who love, who feel great truths and tell them, and the truth of truths is love;" in other words, poetry and sentiment will refine and ennoble you. It is a mistake to suppose that a poet must write. The greatest of all never put pen to paper. What sublimer poetry than the parables of Christ, but they were voiced, not written. All who love the beautiful are more or less poetical. Cultivate the love of the beautiful, and, at the same time, cultivate human sympathy. Do not be afraid to be reasonably sentimental. A hog has no use for sentiment, but then hogs are of no value except for fat food and pepsin. Develop cheerfulness. Develop the disposition to overlook the weaknesses and frailties of man. No one on earth needs more to be forgiving, to be patient, to be kind, to be thoughtful, to be a commander of his own spirit, than the doctor. Never have any grievances. Read your code of ethics and then boil it down and throw the scum and *débris* away and you will have left to you the only code that the profession requires,—the unwritten law which governs gentlemen, and that is all summed up in the Golden Rule, "As ye would that others should do unto you, do ye even so to them." Don't waste your time trying to discover and punish the code breakers. It is a thankless task.

The thought constantly before the doctor should be, "put yourself in his place." Surely, then, duty is more likely to be well done and charity exercised.

Do not believe anything that a patient may say in derogation of another doctor. Above all, give no ear to statements said to have been made by other physicians reflecting upon yourselves. Ninety-nine times out of a hundred, they have been misunderstood and modified in the repeating. Believe nothing disagreeable and unkind that you hear of any one, and very little that you see or know to be true.

It goes without saying that the more you exercise your memory within reason, the more you develop it. Constant effort should be made in this direction. It will be well for you also to cultivate your "forgetter." You will hear many things and have reposed in you many confidences which will affect, in a most sacred and vital way, the interests of others. Form the habit of forgetting these things. This will soon be done if you never repeat them and strive not even to think of them. Discuss the personalities and facts pertaining to your cases nowhere; not even at home to your own wife and family. More doctors are gotten into hot water by an inadvertent word dropped by themselves and repeated, than by any other means.

Of course, there will come a time, and it may be near to many of you, when you will meet her "whose very frowns are fairer far than the smiles of other maidens are." Weigh well your step. The doctor should either be well married or not at all. This, you may say, applies to all, but it applies more to doctors than to others. The well-married doctor, other things being equal, is the better doctor. A man has to be a mighty good man, a very broad-gauged man, and a very strong one, not to become selfish and narrow by living alone. The doctor's wife must have a goodly store of patience, endurance, and amiability, and as the doctor's patients always have to show their tongues, she must religiously never show hers. She must be so thoroughly unselfish that she cannot be jealous, because she will soon learn that her husband belongs more to other people than her.

Remember all things work together for good to the members of the medical profession who love science and keep its commandments; whose hearts beat in sympathy with humanity, and who work without ceasing. In this connection, permit me to say that you fail in your duty to yourself, and to those depending upon you, and to the community, unless you properly develop your business end. In the beginning of your career, be ready always to serve all ; to give your strength and knowledge without money and without price, and if you cannot get something to do otherwise, I would almost say that you are justified in offering premiums for opportunities for serving sufferers. The poor you will find your truest and best friends, and grateful, too, they are ; and the sweetest fee that you will ever receive will be the hearty "God bless you," of the pauper patient. Study your cases ; serve them well, and have in whatever community you may locate, walking advertisements, giving evidence of your skill, and this is the only advertising that you will need. As you advance, make it your habit, in a careful and discriminating way, to be paid for your services where it can be done. Later on, you are justified in declining your services to those who are able to pay and will not. Systematic care will soon enable you to weed out the unworthy and unappreciative. As you grow older in your profession, resolve to develop some special skill. Your tastes will materialize, and you will know in what particular direction you may be considered an expert, and now begin to weed your practice. Get rid of your ambition to have the largest visiting-list in the community. Do less work and better work. Pursuing this course, you need not, as many do in the profession, grow old ungracefully, and with hat in hand anxiously seek patients and make frantic efforts to hold those which you have had, but who are now disposed to go elsewhere. Remember that this is the age for the young man. More and more the world wants activity, drive, alertness, energy, and will have it, and with the progress of years, undoubtedly, this tendency will increase, and it behooves every one of us to realize that the time for us to do our best work, is before we are sixty.

Let us know to the fullest that "When he is forsaken, withered and shaken, there is nought an old doctor can do but die." But we need not be "forsaken, withered and shaken," if we pursue the right course. By mingling with the young in our profession, by cultivating a love for children, Nature, a sympathy for the suffering, we will keep our hearts young, and as we cross over upon the shady side of life's mountain, though the bright and beaming sun be behind us, the nearer we approach the dark valley, the more joyous will be our thoughts, for we will ever be cheered by memories of the past, by singing birds and babbling brooks, by being completely in touch with the young and the gladsome, the good, the true, the beautiful, and the jolly.

Though there may be aches, pains, disappointments, sadness, and clouds, we will have a balm for every pain; something to compensate us for every disappointment; a comfort in others' gladness for our sadness; and beyond the darkness and the clouds, another sun will be shining, and that will be ours, together with a realization of that which is expressed in one of the sweetest words in all our vocabulary—rest.

And now, gentlemen of the graduating class of 1893, on behalf of the Faculty of the Marion-Sims College of Medicine, I bid you a fond farewell.

As the years come and go, may you have all the success and honor, all of the sunshine and good cheer, and all of the sweet home-life you could wish for. "Look not mournfully into the past. It comes not back again. Wisely improve the present; it is thine. Go forth to meet the shadowy future, without fear and with a manly heart," and may God bless you every one, and keep you from harm.

